# Fine Particulate Matter Concentrations in Urban Chinese Cities, 2005–2016: A Systematic Review

**DOI:** 10.3390/ijerph14020191

**Published:** 2017-02-14

**Authors:** Mike Z. He, Xiange Zeng, Kaiyue Zhang, Patrick L. Kinney

**Affiliations:** 1 Department of Environmental Health and Engineering, Johns Hopkins University Bloomberg School of Public Health, Baltimore, MD 21205, USA; 2 Department of Environmental Health Sciences, Columbia University Mailman School of Public Health, New York, NY 10032, USA; 3 Program in Public Health Studies, Johns Hopkins University Krieger School of Arts and Sciences, Baltimore, MD 21218, USA; xzeng5@jhu.edu; 4 Jiangsu Provincial Center for Disease Control and Prevention, Nanjing 210000, Jiangsu, China; m15195969591@163.com; 5 Yangzhou Center for Disease Control and Prevention, Yangzhou 225000, Jiangsu, China; 6 Department of Environmental Health, School of Public Health, Boston University, Boston, MD 02118, USA; pkinney@bu.edu

**Keywords:** air pollution, particulate matter, PM_2.5_, China, systematic review, ambient air

## Abstract

*Background*: Particulate matter pollution has become a growing health concern over the past few decades globally. The problem is especially evident in China, where particulate matter levels prior to 2013 are publically unavailable. We conducted a systematic review of scientific literature that reported fine particulate matter (PM_2.5_) concentrations in different regions of China from 2005 to 2016. *Methods*: We searched for English articles in PubMed and Embase and for Chinese articles in the China National Knowledge Infrastructure (CNKI). We evaluated the studies overall and categorized the collected data into six geographical regions and three economic regions. *Results*: The mean (SD) PM_2.5_ concentration, weighted by the number of sampling days, was 60.64 (33.27) μg/m^3^ for all geographic regions and 71.99 (30.20) μg/m^3^ for all economic regions. A one-way ANOVA shows statistically significant differences in PM_2.5_ concentrations between the various geographic regions (F = 14.91, *p* < 0.0001) and the three economic regions (F = 4.55, *p* = 0.01). *Conclusions:* This review identifies quantifiable differences in fine particulate matter concentrations across regions of China. The highest levels of fine particulate matter were found in the northern and northwestern regions and especially Beijing. The high percentage of data points exceeding current federal regulation standards suggests that fine particulate matter pollution remains a huge problem for China. As pre-2013 emissions data remain largely unavailable, we hope that the data aggregated from this systematic review can be incorporated into current and future models for more accurate historical PM_2.5_ estimates.

## 1. Background

Air pollution has long been considered a major environmental problem around the world, and the concern about particulate matter (PM) has been increasing in the last decades. A number of studies have measured ambient air concentrations of PM in numerous large cities in China, but no study to date has attempted to evaluate these results collectively. Furthermore, although China has officially started to monitor PM_2.5_ levels since 2013, pre-2013 data remains unavailable to the public, making it difficult to study any historical trends of PM_2.5_ in China. This paper aims to gather data collected on fine particulate matter in the scientific literature using a systematic review approach. In doing so, this review has two goals: (1) to quantify and contrast ambient air PM_2.5_ concentrations in six geographic and three economic regions, and (2) to provide a database of pre-2013 measured PM_2.5_ data useful for current and future modelers to calibrate modeling data.

Particulate matter is defined as “a complex mixture of extremely small particles and liquid droplets, made up of acids, organic chemicals, metals, and soil or dust particles” [1]. Particulate pollution is divided into several categories based on its size. Inhalable coarse particles, also known as PM_10_, are particulate matter that has an aerodynamic diameter less than 10 micrometers. Fine particles, or PM_2.5_, are particles that have an aerodynamic diameter less than 2.5 μm. Fine particles are further divided into two refined categories: ultrafine (0.01 μm to 0.1 μm) and fine (0.1 μm to 2.5 μm) particles [1]. For the purposes of this study, we will only distinguish between the two large categories of PM_10_ and PM_2.5_.

The size of particulate matter has been directly linked to its potential of causing health problems, with smaller particles posing a greater threat than larger ones do [1]. Because of its small size, PM_2.5_ actually bypasses the human body’s defense mechanism, and it can lodge deeply into the respiratory system and from there be absorbed into the systemic circulation. With no natural clearance mechanism, the body is unable to efficiently remove PM_2.5_, resulting in an increase in the risk of many cardiorespiratory disorders [2], including pulmonary and systemic oxidative stress, immunological modifications, hypoxemia, atherosclerosis, a faster progression of chronic obstructive pulmonary disease, a decrease in lung function, as well as other cardiovascular diseases [3].

Fine particulate matter has become an increasing environmental problem in China due to the country’s face-paced economic development, rapid urbanization, and drastic increase in the use of motor vehicles. Because PM_2.5_ was not a pollution criterion in China’s National Ambient Air Quality Standards (NAAQS) of 1996, so it was not mandatorily monitored and there had been no official national data on PM_2.5_ emissions up until 2013. China updated its NAAQS in 2012 to include PM_2.5_, and these new standards have become effective as of 1 January 2016 [4].

In their 2014 paper, Yao and Lu estimated particulate matter concentrations in China with remote sensing and discovered spatial differentiation of both PM_10_ and PM_2.5_ in various regions using an artificial neural network (ANN), showing that some regions suffers from more serious PM_2.5_ pollution than PM_10_ pollution [5]. However, the ANN was partially trained using PM_2.5_ data from the United States, which is a large potential source of error for their predictions. Ma et al. (2016) estimates PM_2.5_ data from 2004 to 2013 using a statistical model, but their model is completely reliant on monitoring data from 2013 to 2014 and does not use any actual historical data to calibrate their models, a limitation that they state in their paper [6]. Both models would benefit greatly with the incorporation of historical PM_2.5_ data, even if this data is only available at a small scale or for short time periods.

We emphasize here that the goal of this review is not to provide a representative overview of PM_2.5_ across China. Rather, it aims to fill the gap of unavailable historical data by looking at published scientific literature that independently collected PM_2.5_ data and aggregate them in one place as a resource for future modelers.

## 2. Methods

We conducted an extensive search for scientific literature that measured ambient air PM_2.5_ concentrations in different Chinese cities using databases PubMed, Embase, and China National Knowledge Infrastructure (CNKI). We used the following free text and Medical Subject Headings (MeSH): “PM_2.5_”, “PM_10_”, “air pollution”, “particulate matter”, and “aerosol”. We also used the names of the China’s 34 province-level administrative divisions. The search period was through 14 January 2016.

**Figure 1:**
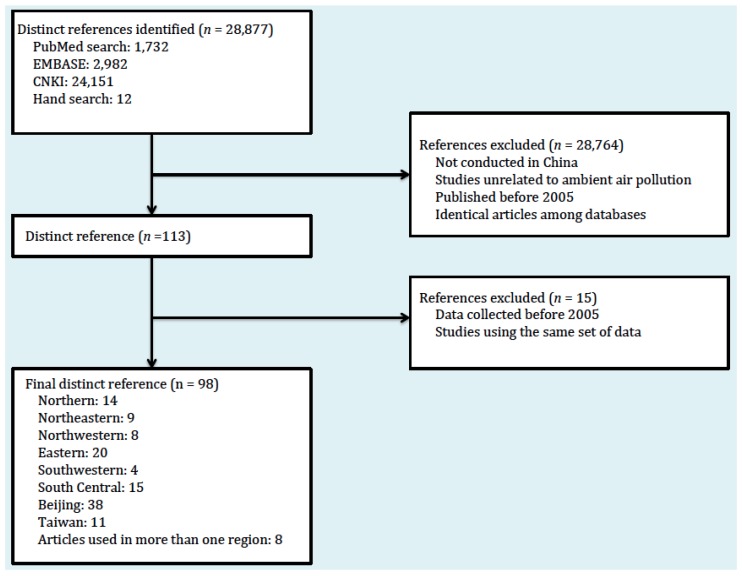
Flow diagram of the study selection process.

**Figure 2:**
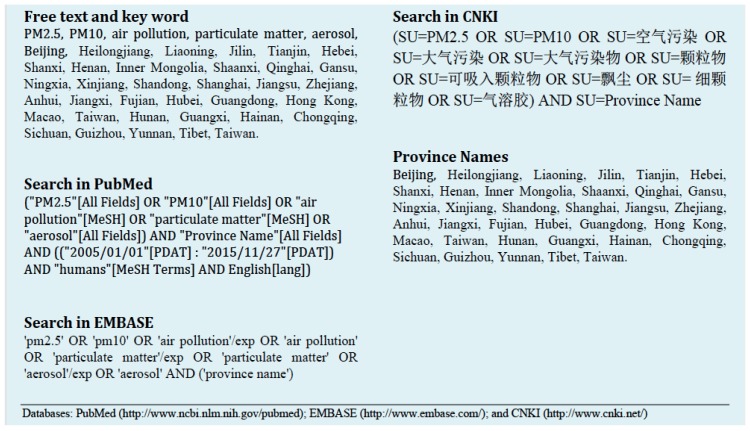
Search strategy.

Only studies that collected original data on ambient PM_2.5_ concentrations in China were included in this review. We excluded studies that are: (1) not conducted in China, (2) unrelated to ambient air pollution levels, (3) analyzing pre-2005 data, and (4) using the same set of data as other cited studies. A flowchart of the study selection process is shown in Figure 1. Applying these exclusion criteria, we identified 98 distinct references including 575 independent measurements from various cities, mostly from provincial capitals and large urban areas. A detailed list of our search strategy is also shown in Figure 2.

**Figure 3:**
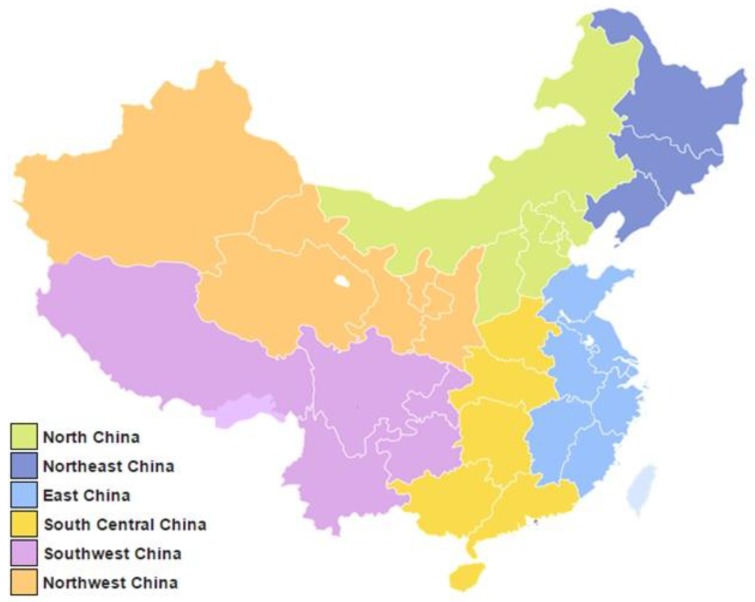
Regions of China.

To facilitate data analysis and allow for adequate comparisons of results, the data were partitioned into six geographically determined regions: Northeastern, Northern, Northwestern, Eastern, South Central, and Southwestern. All of these regions are consistent with China’s official “traditional regions,” which are the current provincial-level divisions grouped by the country’s former administrative areas from 1949 to 1952. A picture of the regions is shown in Figure 3 (adapted from work by Ericmetro, redistribution allowed under Creative Commons Public License CC BY-SA 3.0). These regions serve as units of comparison that are fairly evenly distributed in terms of geographic size and population density, but other equally valid divisions exist as well (for example, dividing the South Central region into separate South and Central regions). Additionally, Beijing was included as a separate category due to the large number of data points, and Taiwan was included as a separate category since it did not belong to the six regions mentioned above.

For policy relevance, we also looked at three important economic regions: the Beijing-Tianjin-Hebei Metropolitan Region, the Yangtze River Delta, and the Pearl River Delta. These regions are consistent with China’s recent “Action Plan on Prevention and Control of Air Pollution”, labeled as the three regions of highest priority over the next five to ten years [7].

Statistical analysis was performed using Stata13 (StataCorp Inc., College Station, TX, USA). Weighted averages of PM_2.5_ concentrations were obtained based on the number of days of sampling in each study. These weights were determined using the number of days of sampling in each study divided by the number of days of sampling in each region. For studies where exact sampling day was not available, sampling months and seasons were used to approximate the number of sampling days. Whenever available, instruments used for sampling and sampling methodology are detailed in the supplementary materials (Supplementary S1 Data.xlsx, S2 Codebook.docx). For the purposes of the study, we categorized March, April, and May as Spring, June, July, and August as Summer, September, October, and November as Autumn, and December, January, and February as Winter. Weighted regional and overall averages are presented in the results section. The results are also compared to China’s current annual standard (35 μg/m^3^) and 24-h standard (75 μg/m^3^) limits for 24-h averages PM_2.5_ concentrations [8]. All PM_2.5_ measurements in this study are reported as μg/m^3^, and measurements from references that are reported in different units are converted accordingly.

## 3. Results

Data was obtained for all 34 of China’s province-level administrative divisions, including 22 provinces, five autonomous regions, four municipalities, two special administrative regions (Hong Kong and Macau), and Taiwan. For the purposes of this study, we will disregard the specific classifications and focus solely on province-level administrative regions. We identified a total of 574 separate measurements: 61 in the Northeastern Region [9,10,11,12,13,14,15,16,17], 53 in the Northern Region [14,17,18,19,20,21,22,23,24,25,26,27,28,29], 42 in the Northwestern Region [14,17,30,31,32,33,34,35], 121 in the Eastern Region [14,17,30,33,36,37,38,39,40,41,42,43,44,45,46,47,48,49,50,51], 136 in the South Central Region [14,17,33,34,52,53,54,55,56,57,58,59,60,61,62], 51 in the Southwestern region [14,17,63,64], 98 in Beijing [10,14,17,19,22,30,33,65,66,67,68,69,70,71,72,73,74,75,76,77,78,79,80,81,82,83,84,85,86,87,88,89,90,91,92,93,94,95], and 12 in Taiwan [96,97,98,99,100,101,102,103,104,105,106].

The Beijing-Tianjin-Hebei metropolitan region, Yangtze River Delta, and Pearl River Delta are all defined in the dataset based on the major cities that make up each of these regions. We identified 123 separate measurements for the Beijing-Tianjin-Hebei metropolitan region, 59 for the Yangtze River Delta, and 38 for the Pearl River Delta.

**Figure 4:**
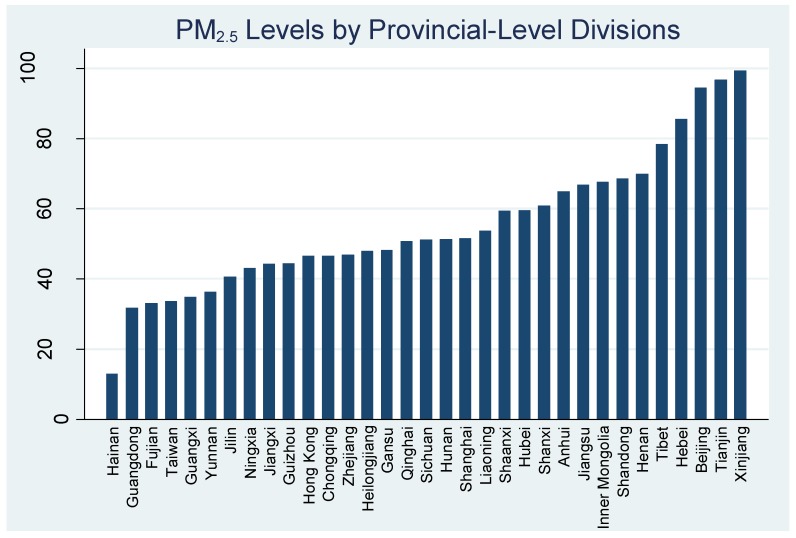
PM_2.5_ levels in different regions.

Figure 4 presents the PM_2.5_ levels in all 34 province-level administrative divisions, and Table 1 summarizes the regional averages reported with their respective standard deviations. The “Number of Measurements” column refers to the number of data points used in each region. The “% Above Annual Limit” column refers to the percentage of data points in each region that exceeded China’s current annual PM_2.5_ emissions limit of 35 μg/m^3^ in urban areas. The “% Above 24-h Limit” column refers to the percentage of data points in each region that exceeded China’s current 24-h PM_2.5_ emissions limit of 75 μg/m^3^ in urban cities. The overall average represents an arithmetic average of all 574 data points weighted by the total number of days of sampling across all regions.

Table 2 provides summaries similar to that of Table 1 but for the three economic regions. Note that there is overlap between the geographic and economic regions. While the geographic regions include all data points collected from the search, the three economic regions only encompasses about 40% of this data. A one-way analysis of variance test of PM_2.5_ concentrations yielded a statistically significant difference (F = 14.91, *p* < 0.0001) among all geographic regions and all economic regions (F = 4.55, *p* = 0.01).

## 4. Discussion

### 4.1. Comments on Findings

As mentioned at the beginning of the paper, the reasoning behind the choice of geographic regions is for the facilitation of data interpretation. The six regions presented in this study are frequently used categories representing the provincial-level divisions in China and this facilitates communication. We decided to report Beijing separately from the Northern Region due to the large amount of current literature we found that reported PM_2.5_ data for Beijing. In fact, Beijing contributed to 98 of the 151 total results from the Northern Region, which would severely bias the results in the Northern Region if analyzed together.

In contrast, the economic regions represent a more policy-relevant division for China’s most critically polluted areas. The Beijing-Tianjin-Hebei metropolitan region in particular has the highest levels of PM_2.5_ of the three economic regions, and the three economic regions overall has higher PM_2.5_ levels than the average of all geographic regions.

Our findings in Figure 4 are slightly different from data available from current monitoring stations in China. Several reasons may contribute to this. First, our data is aggregated from a variety of data sources, each with different sampling methodologies. Furthermore, data collection from these sources are usually focused on select large cities, leaving hundreds of other cities in the provinces excluded due to lack of data. Lastly, many data points from this study were collected prior to 2013, back when a national PM_2.5_ monitoring network was not yet existent. These data points were also used for the averages provided in Figure 4 and contribute to potential discrepancies.

One of the most challenging parts of this study was standardizing the dataset for proper analysis. With numerous papers that recorded data at different times of the year using different timeframes and different instruments, it was necessary to come up with a methodology to allow the maximum number of data to be interpretable. Most papers reported PM_2.5_ in μg/m^3^, which is a standard measure of particulate matter and other compounds. Data with different units were converted to μg/m^3^ for analysis. The sample size was determined based on the number of days of sampling, which was either directly stated or implied in each paper included in this study. The presence of a sample size allowed for proper adjustment of the data, allocating more weight to studies that made measurements for a longer period of time than those with shorter timeframes. This step was crucial for the analysis, as papers included in this study collected samples for as short as three days and as long as several years.

As seen in Table 1, the Northwestern Region had the highest mean levels of PM_2.5_ at 85.4 μg/m^3^, followed by the Northern and Northeastern regions. Beijing had the highest mean levels of PM_2.5_ at 94.42 μg/m^3^, while Taiwan had the lowest mean levels at 30.49 μg/m^3^. The fact that Beijing had the highest levels out of all considered categories shows that we were right to consider it as a separate category, as it had the potential to bias the results in the Northern Region if left unaddressed. In contrast, while Taiwan had the fewest number of observations (n = 12), it produced both the lowest concentrations and the smallest standard deviation, showing the consistency of the measurements that were taken in various cities.

We chose to use both the annual and the 24-h PM_2.5_ standards as a means to be as conservative as possible with our estimates. The vast majority of the gathered data points were taken from large urban cities, to which these standards apply. We see from Table 1 that based on the annual standard, nearly 90% of all data points exceeded the limit. Even under the most lenient standard in the nation, 32% of all data points still exceed the limit. In fact, China’s standards are already very lenient. For reference, the World Health Organization guidelines for PM_2.5_ is only 10 μg/m^3^ for the annual mean and 25 μg/m^3^ for the 24-h mean, approximately three times lower than that of China’s permitted levels [107]. These results are a strong indicator that most if not all large cities in China suffer from air pollution and especially particulate matter pollution. Undoubtedly, China’s PM_2.5_ problem is severe, and a long-term commitment is required to resolve it.

### 4.2. Applications to Current Literature

There have been a number of related studies published in the literature recently. Krzyzanowski et al. looked at annual average concentrations of PM_2.5_ in a number of mega-cities from around the world, including 12 cities from China [108]. Krzyzanowski used both surface monitoring and modeling used by the Global Burden of Disease 2010 [109] to estimate particulate matter exposure. GBD 2010 utilized a combination of satellite estimates and high resolution air quality models to complement surface monitoring data, allowing for estimates to be made in areas even where surface monitoring data were unavailable [108]. This is an approach that should be seeing more use in the future as modeling technology continues to advance at an incredible speed.

A similar study was conducted by Yao and Lu in 2014 which focused on looking at particulate matter and population exposure in China using remote sensing techniques. Yao and Lu used three types of data: atmospheric aerosol product derived from NASA, meteorological data for atmospheric analysis, and population density data from the Center for International Earth Science Information Network (CIESIN) [5]. Using an ANN algorithm that was suggested in a prior paper by Gupta et al. [110], Yao and Lu built a framework centered on the ANN called the ANN estimation model, which they then used to estimate particulate matter concentration. While the methods of Yao and Lu are innovative, their ANN was partially trained using PM_2.5_ data from the United States, a potential source of substantial error for their predictions. Their model would benefit from the incorporation of collected data, which our paper provides to a certain extent.

More recently (2016), Ma et al. reconstructed historical PM_2.5_ data from 2004 to 2013 using a combination of aerosol optical depth data and a two-stage statistical model [6]. Although their model shows promising results at the seasonal and monthly levels, it is based completely on extrapolating newer data (2013–2014) with no calibration or comparison to actual data from the earlier time periods. The authors note this as one of their major limitations and notes that historical PM_2.5_ data would allow them to be able to make annual model adjustments. Once again, the data aggregated from this review, albeit mostly short-term and relatively few in numbers, may be incorporated into their model for more accurate estimates.

### 4.3. Limitations

There are several limitations to our study. Two types of variation were detected in our study: seasonal and regional (urban versus suburban areas). Seasonal variation was one of the challenges we expected to face in our study. Wang et al. was one of the few papers that attempted to quantify differences in PM_2.5_ fluctuations over the seasons, and noticed that PM_2.5_ concentrations were highest in the winter, following a trend of winter > autumn > spring > summer [59]. Using our formal definition of season, we attempted to categorize all obtained data points into a season if possible. This was not possible for annual data or data that were reported collectively over more than one season (e.g., March to August). A large portion of our dataset did come from the wintertime, making the results an overestimate of annual PM_2.5_ concentration but probably an underestimate of winter PM_2.5_ concentration. Please see the supplemental files for more details.

Regional variation was a smaller and easier problem to manage. A number of papers separated collected data for urban and suburban areas. In general, urban areas were found to have slightly higher concentrations of PM_2.5_ [56,59,111]. For papers that reported urban and suburban averages separately, the two numbers were reported as separate entries based on the number of sampling days.

Lastly, differing measurement methods and sampling locations pose a potential threat to the validity of our results. The data used from this review comes from a variety of studies that collected PM_2.5_ for various reasons. These studies often used different instruments to collect PM_2.5_ and collected data from different areas of cities (e.g., residential areas versus highways). Whenever available, we included the instrument and method used for data collection in our Supplementary materials, but detailed sampling locations were generally not available.

## 5. Conclusions

This analysis provides evidence that based on existing research, PM_2.5_ levels vary among different geographic and economic regions of China. More importantly, this review fills the gap of pre-2013 ambient PM_2.5_ concentrations in China, which is unavailable to the public, with PM_2.5_ data collected independently by different studies. Until widespread pre-2013 PM_2.5_ data for China becomes available, the aggregated dataset from this review may be used as a starting point for calibrating and improving current and future air pollution models.

## Figures and Tables

**Table 1:** Summary of geographic regions.

Region	[PM_2.5_] (μg/m^3^)	Number of Measurements	% Above Annual Limit ^1^	% Above 24-h Limit ^2^
Northeastern	66.50 ± 27.96	61	91.80%	34.43%
Northern	76.10 ± 38.69	53	100%	50.94%
Northwestern	85.41 ± 59.19	42	100%	14.29%
Eastern	55.41 ± 18.16	121	86.78%	20.66%
South Central	50.23 ± 21.00	136	75.74%	16.91%
Southwestern	48.72 ± 13.63	51	90.20%	11.76%
Beijing	94.42 ± 23.83	98	100%	77.55%
Taiwan	30.49 ± 1.81	12	8.33%	0%
**Overall Average**	**60.64 ± 33.27**	**574**	**87.80%**	**32.06%**

^1^ Annual limit is 35 μg/m^3^. ^2^ 24-h limit is 75 μg/m^3^.

**Table 2:** Summary of three economic regions.

Region	[PM_2.5_] (μg/m^3^)	Number of Measurements	% Above Annual Limit ^1^	% Above 24-h Limit ^2^
BTH	93.73 ± 25.89	123	100%	78.05%
Yangtze River	55.86 ± 17.62	59	93.22%	28.1%
Pearl River	47.23 ± 14.86	38	65.79%	13.16%
**Overall Average**	**71.99 ± 30.20**	**220**	**92.27%**	**53.64%**

^1^ Annual limit is 35 μg/m^3^. ^2^ 24-h limit is 75 μg/m^3^.

## Supplementary Materials

The following are available online at www.mdpi.com/1660-4601/14/2/191/s1, Supplementary S1: The complete dataset of all data used for this systematic review. Supplementary S2: Codebook: A word document that defines the names used in the Supplementary materials.
