# Supplementary material for: Fine Particulate Matter Concentrations in Urban Chinese Cities, 2005–2016: A Systematic Review
Source: Int J Environ Res Public Health. 2017 Feb 14;14(2):191. doi: 10.3390/ijerph14020191 (PMC5334745; doi:10.3390/ijerph14020191)
Supplement: Supplementary file 1 [file ijerph-14-00191-s001.zip › ijerph-176239-supplementary-done/S2 Codebook.pdf]

Supplementary Materials: Fine Particulate Matter Concentrations in Urban Chinese Cities, 2005–2016: A Systematic Review

Mike Z. He, Xiange Zeng, Kaiyue Zhang and Patrick L. Kinney

Codebook for Supplemental Data
Author – author(s) of the study that the data was extracted from
Region – represents six possible geographic regions (Northeastern, Northern, Northwestern, Eastern, South Central, and Southwestern), Beijing, and Taiwan
City – city that extracted data came from
YearPublished – article's year of publication
YearCollected – year(s) that data used in the study was collected from
MonthDate – specific months/dates for data collection (when available); data collected over entire year or multiple years are marked as "Annual"
Season – denoted as Spring (March-May), Summer (June-August), Autumn (September-November), and Winter (December-February)
PM25gm3 – concentration of $PM_{2.5}$, measured in $\mu g/m^3$
SD – standard deviation of PM25gm3
SampleSize – number of sampling days in the city where data is collected
Hour/Annual – dichotomous variable defining data as 24-hour or annual data
Exceed35Limit – dichotomous variable on whether data point exceeds China's annual $PM_{2.5}$ limit of $35 \mu g/m^3$ (0=no, 1=yes)
Exceed75Limit - dichotomous variable on whether data point exceeds China's 24-hour $PM_{2.5}$ limit of $75 \mu g/m^3$ (0=no, 1=yes)
DataCollectionMethods – notes describing the data source, methods, and instruments used for data collection, whenever available
